# Innate B-1 B Cells Are Not Enriched in Red Blood Cell Autoimmune Mice: Importance of B Cell Receptor Transgenic Selection

**DOI:** 10.3389/fimmu.2017.01366

**Published:** 2017-11-03

**Authors:** Amanda L. Richards, Heather L. Howie, Linda M. Kapp, Jeanne E. Hendrickson, James C. Zimring, Krystalyn E. Hudson

**Affiliations:** ^1^Bloodworks Northwest Research Institute, Seattle, WA, United States; ^2^Department of Laboratory Medicine and Pediatrics, Yale University, New Haven, CT, United States; ^3^Department of Laboratory Medicine, Division of Hematology, University of Washington, Seattle, WA, United States; ^4^Department of Internal Medicine, Division of Hematology, University of Washington, Seattle, WA, United States

**Keywords:** B cell tolerance, red blood cells, autoimmunity, autoimmunity models, autoantibodies

## Abstract

Autoimmune hemolytic anemia (AIHA) results from breakdown of humoral tolerance to RBC antigens. Past analyses of B-cell receptor transgenic (BCR-Tg) mice that recognize RBC autoantigens led to a paradigm in which autoreactive conventional B-2 B cells are deleted whereas extramedullary B-1 B cells escape deletion due to lack of exposure to RBCs. However, BCR-Tg mice utilized to shape the current paradigm were unable to undergo receptor editing or class-switching. Given the importance of receptor editing as mechanism to tolerize autoreactive B cells during central tolerance, we hypothesized that expansion of autoreactive B-1 B cells is a consequence of the inability of the autoreactive BCR to receptor edit. To test this hypothesis, we crossed two separate strains of BCR-Tg mice with transgenic mice expressing the BCR target on RBCs. Both BCR-Tg mice express the same immunoglobulin and, thus, secrete antibodies with identical specificity, but one strain (SwHEL) has normal receptor editing, whereas the other (IgHEL) does not. Similar to other AIHA models, the autoreactive IgHEL strain showed decreased B-2 B cells, an enrichment of B-1 B cells, and detectable anti-RBC autoantibodies and decreased RBC hematocrit and hemoglobin values. However, autoreactive SwHEL mice had induction of tolerance in both B-2 and B-1 B cells with anti-RBC autoantibody production without anemia. These data generate new understanding and challenge the existing paradigm of B cell tolerance to RBC autoantigens. Furthermore, these findings demonstrate that immune responses vary when BCR-Tg do not retain BCR editing and class-switching functions.

## Introduction

Establishment and maintenance of immunological tolerance is essential to prevent autoimmunity, and breakdown of tolerance to red blood cell (RBC) antigens may lead to autoantibody production. Development of anti-RBC autoantibodies can occur secondary to lymphoproliferative disorders, infections, or blood transfusions (1, 2); however, in some cases, autoantibodies to RBCs appear to be the result of primary immunodysregulation (3). The process of autoantibody generation to RBC antigens occurs much more frequently than is generally appreciated, since the vast majority of RBC-specific autoantibodies do not cause any detectable hemolysis or illness. Indeed, up to one out of every 1,000 asymptomatic healthy blood donors have detectable autoantibodies specific for RBCs. However, in the instances where RBC autoantibodies do induce hemolysis, then autoimmune hemolytic anemia (AIHA) can ensue, leading to a severe and sometimes fatal disease (4).

Our understanding of the mechanisms by which B cell tolerance to self-antigens is established and maintained has been significantly advanced through the use of B-cell receptor transgenic (BCR-Tg) mice (5–8). Detailed analyses of BCR-Tg systems have established that developing B cells are tolerized to self-antigens in the bone marrow through receptor editing, deletion, and/or anergy. Applying this approach to the study of B cells specific for autoantigens on RBCs has been carried out in a series of elegant studies using the autoAb 4C8 BCR-Tg mouse model, in which transgenic mice carry immunoglobulin (Ig) genes derived from an anti-erythrocyte autoantibody, 4C8 (9). AutoAb 4C8 BCR-Tg mice show a relative absence of autoreactive conventional (B-2) B cells in the bone marrow and secondary lymphoid organs. By contrast, autoreactive B-1 B cells are enriched in the peritoneal cavity and lamina propria (10, 11). Experimental introduction of a B cell mitogen induces expansion of autoreactive B-1 B cells, correlating with increased autoantibody secretion (12). As a consequence of autoantibody production and hemolysis, the autoAb 4C8 BCR-Tg mice become anemic. Autoantibodies and disease pathology are resolved after elimination of B-1 B cells through hypotonic shock or antigen-induced cross-linking of the BCR (13). Together, these data lead to a model in which B-1 B cells represent a dangerous population in which BCRs specific for RBC autoantigens are not tolerized, presumably because B-1 B cells can develop in extramedullary spaces in which RBC antigens are not likely to be encountered. These data are the basis for the prevailing paradigm of B cell tolerance to RBC autoantigens.

Although the autoAb 4C8 BCR-Tg mouse model led to substantial generation of new knowledge, there are several limitations. The transgenic BCR of the autoAb 4C8 mouse recognizes Band 4.1 (14), a ubiquitous RBC antigen required for RBC membrane stability. Band 4.1 knockout mice are viable (15, 16); however, they exhibit moderate AIHA and their unstable RBC membranes result in abnormal morphology. As such, Band 4.1 knockout mice are not a good control to analyze the effects of the anti-RBC B cells in the absence of autoantigen in the autoAb 4C8 model. In addition, autoAb 4C8 BCR-Tg mice were designed with random integration, are genetically restricted to IgM, and cannot undergo BCR rearrangement, receptor editing, or class-switching. Finally, while the severe hemolysis and chronic inflammation resulting from autoantibody production models AIHA pathology, the presence of autoantibodies simultaneously complicate analysis of baseline immunology. To circumvent these limitations, we engineered a novel model of B cell tolerance to an RBC antigen by utilizing HOD transgenic mice. The HOD mouse expresses a triple fusion protein consisting of hen egg lysozyme (HEL), ovalbumin, and human blood group antigen Duffy (HOD), driven by an RBC-specific promoter (17). Similar to many human RBC autoantibodies, antibodies specific for the HOD antigen do not promote hemolysis.

Two separate BCR-Tg mice with specificity for HEL (contained within HOD) have been described (7, 18). IgHEL mice are random transgenic animals with heavy and light chains specific for HEL, and like autoAb 4C8 BCR-Tg mice, are incapable of undergoing BCR rearrangement, receptor editing, or normal class-switching (7). By contrast, the SwHEL mice were generated with the same Ig transgene as the IgHEL mice, but the heavy chain VDJ region was inserted into the Vh10 locus by homologous recombination; the same light chain was used in both IgHEL and SwHEL animals (18). In this way, anti-HEL B cells in the SwHEL mouse can undergo BCR editing in the bone marrow and can participate in germinal center reactions that result in class-switching to each of the natural antibody isotypes. To further study the mechanisms of B cell tolerance to an RBC autoantigen, we bred HOD mice with either IgHEL or SwHEL mice. This approach allows a direct juxtaposition of the experimental effects of allowing (or preventing) BCR receptor editing, recombination, and class-switching of an Ig specific for an RBC autoantigen.

## Results

### Peritoneal B-1 B Cells Are Enriched in Autoreactive IgHEL But Not SwHEL Mice

**Figure 1:**
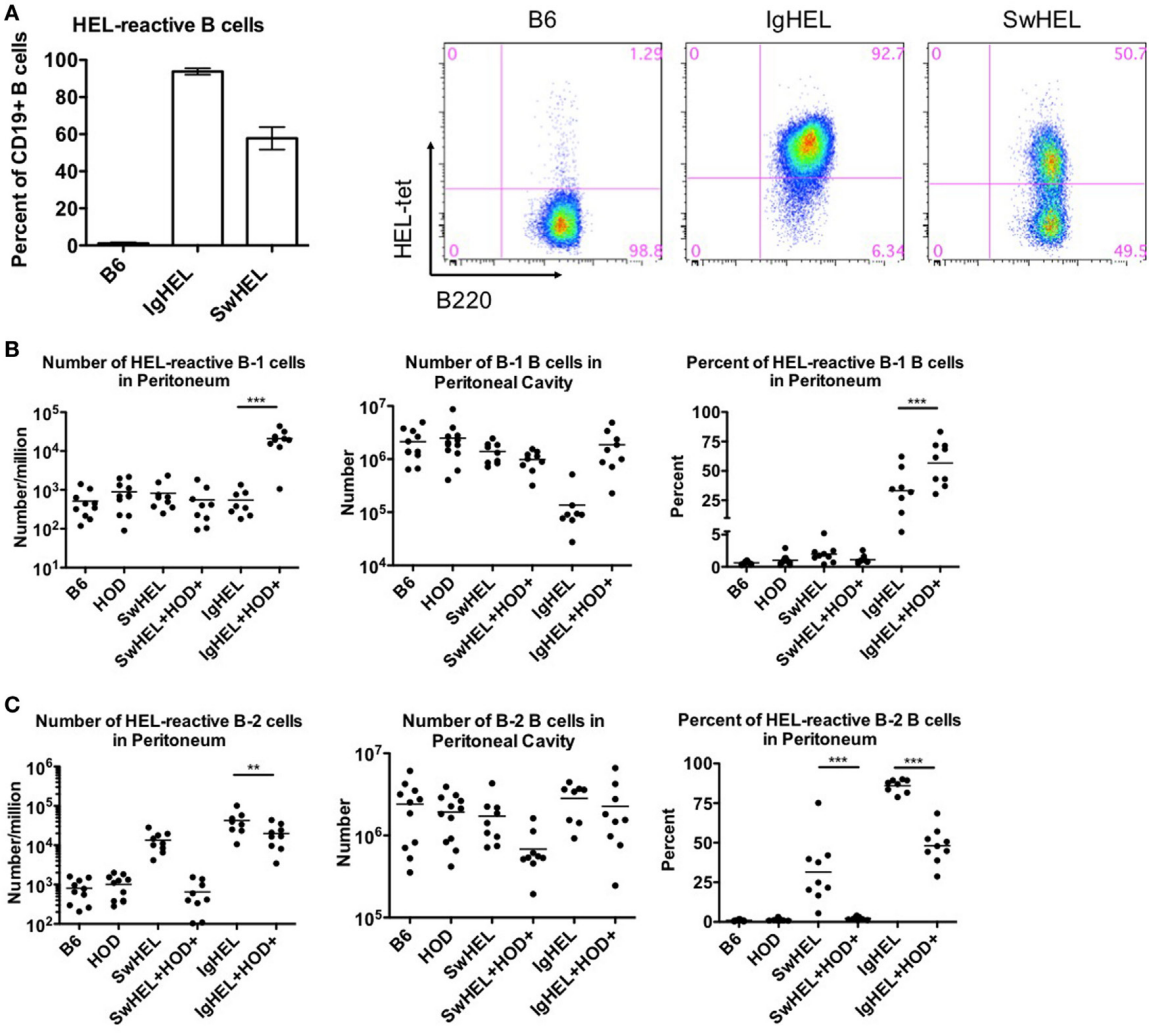
B-1 B cells are enriched in autoreactive IgHEL+HOD+ but not SwHEL+HOD+. Splenocytes and peritoneal cells were harvested and evaluated for expression of cell surface markers to determine the number, frequency, and phenotype of hen egg lysozyme (HEL)-reactive B cells. **(A)** Splenocytes from B6 (left), IgHEL (middle), and SwHEL (right) mice were stained with anti-CD19, anti-B220, and fluorescently labeled HEL (HEL-tet) to determine the frequency of HEL-reactive B cells. Data are representative data from three independent experiments (with at least three mice per group), bar graphs are mean ± SD. Bar graphs and flow plots are gated on CD19+ cells. Peritoneal cells were stained with anti-CD19, anti-IgM, anti-CD43, anti-IgD, and HEL-tet to delineate **(B)** B-1 and **(C)** B-2 B cell subsets and to determine reactivity with HEL. Data shown in **(B,C)** are compiled from three independent experiments with at least three mice per group. Selective *p* values are shown on graphs and * ≤ 0.05, ** ≤ 0.01, and *** ≤ 0.001. For complete statistical analysis with all significant differences, see Table S1 in Supplementary Material.

Both IgHEL and SwHEL mice are on a C57BL/6 (B6) background and both were generated using the HyHEL10 heavy and light chain genes, which confer specificity for HEL (6, 18). Accordingly, BCR-Tg B cells found in either IgHEL or SwHEL mice express a BCR with the same HEL-specific paratope. To track and enumerate HEL-reactive B cells in either IgHEL or SwHEL mice, cells were stained with anti-B220 and tetramerized-HEL-APC (HEL-tet). Consistent with previously published data (18, 19), over 90% of B cells from IgHEL mice and 40–60% of B cells in SwHEL mice reacted with HEL-tet (Figure 1A, left and representative flow plots, right).

Previous data with the autoAb 4C8 BCR-Tg mouse model provided evidence that autoantibodies were a consequence of incomplete tolerance in the B-1 B cell compartment in the peritoneal cavity (10). To test the association of peritoneal autoreactive B-1 B cells in tolerance to RBC-specific autoantigens, both IgHEL and SwHEL mice were crossed with HOD mice, whereby HEL is part of the HOD fusion construct that has RBC-specific expression (20). B-1 B cells were defined as CD19+IgM+CD43+ events whereas B-2 B cells were defined as CD19+IgM+IgD+CD43− events. HEL-reactive B cells in these populations were determined by binding to HEL-tet.

Control B6 mice had fewer than 1,000 HEL-reactive B-1 B cells detectable in the peritoneum, representing the normal background staining for these mice (Figure 1B, left panel; Table S1 in Supplementary Material). No significant difference in this signal was observed in HOD, SwHEL, or IgHEL mice; thus, neither the presence of the HOD antigen nor a HEL-specific Ig transgene increased the number of HEL-reactive B-1 B cells in peritoneal cavity. Co-expression of the Ig transgene and the cognate autoantigen (HEL) in the IgHEL+HOD+ and SwHEL+HOD+ mice yielded different observations; the number of HEL-reactive peritoneal B-1 B cells was similar between SwHEL and autoreactive SwHEL+HOD+ mice; however, unlike the observations made with SwHEL animals, there was a significant increase in HEL-reactive B-1 B cell numbers in IgHEL+HOD+ mice, compared to the IgHEL mice (Figure 1B, left panel; Table S1 in Supplementary Material).

The observed increase of HEL-reactive B-1 B cells in IgHEL+HOD+ mice was not due to a general increase in B-1 B cells, as the absolute number of peritoneal B-1 B cells (of any specificity) was not increased in IgHEL+HOD+ mice compared to other groups (Figure 1B, middle panel). On the contrary, a 10-fold decrease in absolute numbers of B-1 B cells was observed in IgHEL mice, compared to control strains; something not observed in SwHEL mice (Figure 1B, middle panel). However, within the decreased B-1 population in IgHEL mice, there was substantial enrichment in the percentage of B cells that were HEL-specific (Figure 1B, right panel), thus accounting for the decrease in total number of B-1 B cells but not in the number of HEL-specific B cells in IgHEL mice. Together, these data indicate that expression of the anti-HEL IgM Ig in the IgHEL mouse (in the absence of the HEL antigen) decreases total B-1 B cell numbers, but the surviving population has a high percentage of HEL-specific B cells. Furthermore, co-expression of HEL with the IgHEL BCR (IgHEL+HOD+ mice) resulted in significantly higher numbers of HEL-reactive peritoneal B-1 B cells. Thus, for the IgHEL mouse, autoantigen promotes the expansion of autoreactive peritoneal B-1 B cells, consistent with the data obtained with the autoAb 4C8 mouse model.

Analysis of the SwHEL mouse gave a very distinct outcome compared to IgHEL. On its own, the SwHEL mouse was not significantly different from the B6 mice with regard to number of B-1 B cells (total or HEL-specific) in the peritoneum, with only a slight (but non-significant) increase in percentage of HEL-specific B-1 B cells (Figure 1B). In stark contrast to the IgHEL mouse, crossing SwHEL with HOD mice failed to significantly alter the number or percentage of B-1 B cells, either HEL specific or total. Moreover, the small increase in HEL-specific B-1 B cells in SwHEL mice was not increased by presence of HOD autoantigen (as observed in IgHEL+HOD+), but was decreased to baseline levels seen in control mice (Figure 1B, right panel). Together, these data draw a sharp distinction between the fates of autoreactive B-1 B cells in IgHEL vs. SwHEL mice, with a preferential increase in autoreactive peritoneal B-1 B cells in IgHEL+HOD+ but not SwHEL+HOD+ animals.

Analysis of B-2 B cells demonstrated that both B6 and HOD mice have similar numbers and percentages of B-2 B cells in the peritoneal cavity, both for absolute numbers and HEL-specific B-2 B cells (Figure 1C, left and middle panels). Consistent with carrying an anti-HEL Ig transgene, both the SwHEL and IgHEL mice have increased B-2 B cells specific for HEL (numbers and percentages) (Figure 1C, left and right panels), but in neither instance is this simply the result of increased total numbers of B-2 B cells as numbers of total B-2 B cells is unaltered in SwHEL or IgHEL mice compared to control strains (Figure 1C, middle panel). Crossing IgHEL or SwHEL with HOD gave distinct results. Numbers of HEL-reactive B-2 B cells were significantly reduced in IgHEL+HOD+ mice compared to IgHEL alone; however, despite this decrease, there remain a significantly elevated number of HEL-reactive B-2 B cells in IgHEL+HOD+ mice compared to controls (Figure 1C, left panel). By contrast, in SwHEL+HOD+ mice, HEL-reactive B-2 B cells were decreased (although this reduction was not statistically significant), returning to control baseline numbers. Some of the decrease in HEL-reactive B-2 B cells in SwHEL+HOD+ mice may be due to a reduction in the total number of B-2 B cells (Figure 1C, middle panel); however, analysis of percentages of HEL-reactive B-2 B cells shows a large percentage of HEL-reactive B-2 B cells in SwHEL mice that were absent in SwHEL+HOD+ animals. A similar trend was observed in the percentage of B-2 B cells that are HEL-reactive in IgHEL+HOD+ mice, but to a much lesser extent.

In aggregate, these findings demonstrate a significant enrichment of RBC autoreactive B-1 B cells in IgHEL+HOD+ mice that is absent in SwHEL+HOD+ mice. Moreover, RBC autoreactive B-2 B cells largely persisted in IgHEL+HOD+ mice, but were essentially absent in SwHEL+HOD+ mice. Thus, the question of how RBC autoantigens educate autoreactive B cells yields a very different answer depending upon whether the BCR transgene is a random integrant that cannot undergo BCR recombination, receptor editing, or class-switching vs. a knock-in that retains the ability to participate in these processes.

### B Cell Development in the Bone Marrow Is Altered in IgHEL BCR-Tg Mice

**Figure 2:**
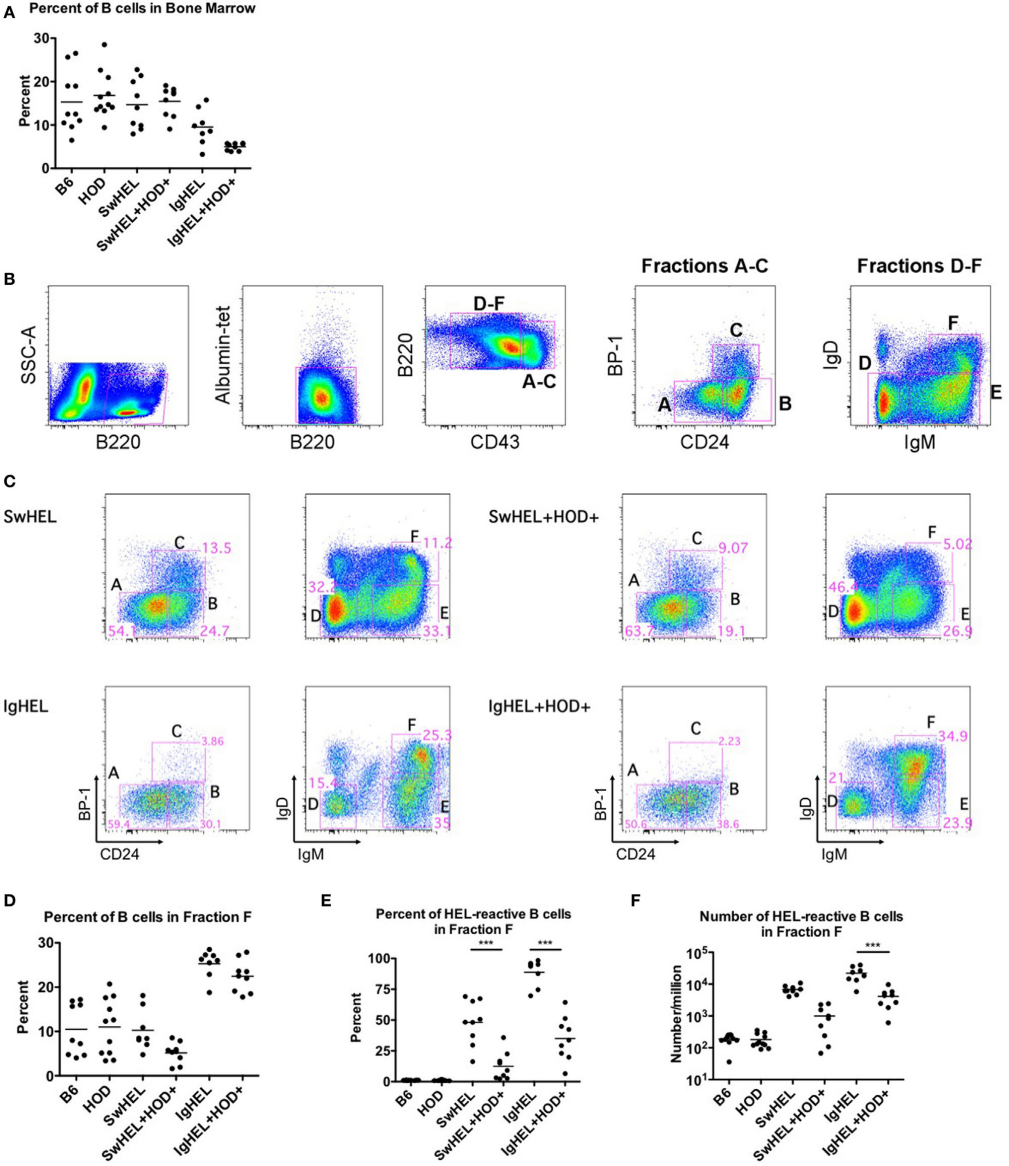
Effect of red blood cell (RBC) autoantigen on B cell development in bone marrow. Bone marrow was harvested, RBCs were lysed, and residual cells were stained with anti-B220, anti-CD43, anti-CD24, anti-IgD, anti-IgM, anti-BP-1, HEL-tet, and control Albumin-tet to delineate the stages of bone marrow development. **(A)** The percentage of B cells in bone marrow was determined for each indicated genotype. **(B)** Bone marrow cells were evaluated for cell surface markers to determine the stages of B cell development, and **(C)** Fractions A–F are shown for each indicated genotype. **(D)** The percent of total B cells, and **(E)** percent and **(F)** number of hen egg lysozyme (HEL)-reactive B cells were determined for Fraction F, the final stage of bone marrow development. Flow plots are representative data from three independent experiments with at least three mice per group. Scatter dot plots in **(A,D–F)** are compiled data from three independent experiments with at least three mice per group. Selective *p* values are shown on graphs and * ≤ 0.05, ** ≤ 0.01, and *** ≤ 0.001. For complete statistical analysis with all significant differences, see Table S2 in Supplementary Material.

To test the effects of RBC-specific antigen expression upon central tolerance mechanisms, B cell development in bone marrow was analyzed by flow cytometry. The baseline percentages of B cells in SwHEL mice was equivalent to B6 and HOD control strains, whereas a slight decrease was observed in IgHEL mice (Figure 2A; Table S2 in Supplementary Material). SwHEL+HOD+ had no decrease in B cell percentages in bone marrow compared to SwHEL mice; by contrast, IgHEL+HOD+ mice had a significant decrease in B cells compared to control B6 and HOD mice and a slight reduction compared to IgHEL+ mice (Figure 2A; Table S2 in Supplementary Material). Thus, the presence of autoantigen results in a decrease in B cell percentages for IgHEL+HOD+ but not SwHEL+HOD+ mice.

To analyze individual stages of B cell development, total B220+ cells were categorized into developmental Fractions A–F using surface expression of B220, CD43, BP-1, CD24, IgD, and IgM. Stages of B cell development consist of A = pre–pro-B, B = pro-B, C = early pre-B, D = IgM − IgD− late pre-B cells, E = new B, and F = antigen naïve, mature B (gating strategy shown in Figure 2B) (21, 22). Percentages of B cells in bone marrow from SwHEL mice (Figure 2C, top row) had similar frequencies of B cells in each developmental stage as was observed in control B6 and HOD mice (data not shown). By contrast, IgHEL mice had a significantly smaller percentage of B cells in Fraction C, the developmental stage for rearrangement of the heavy chain locus (Figure 2C, bottom row). In addition, IgHEL mice had a decrease in the percentage of B cells detected in Fraction D (pre-B cells with phenotype IgM − IgD−), during which light chain rearrangement occurs. Concomitantly, IgHEL mice had a significant increase in the percentage of cells detected in Fraction F, the final stage of B cell maturation where B cells express IgM+IgD+. Accordingly, IgHEL and SwHEL mice have different baseline B cells at each stage of development (in the absence of autoantigen), likely as a result of the different genetic constructs.

Bone marrow from IgHEL and SwHEL mice was juxtaposed with IgHEL+HOD+ and SwHEL+HOD+, respectively, to determine the effects of RBC autoantigen expression on B cell development (representative flow plots are shown in Figure 2C, right panels). Whereas B6, HOD, and SwHEL mice had a similar percentage of B cells in fraction F (mature B cells), a significant increase was observed in IgHEL mice (Figure 2D). The presence of autoantigen resulted in a 2-fold decrease in the percentage of B cells in Fraction F in SwHEL+HOD+ mice compared to SwHEL+ animals (Figure 2D). By contrast, IgHEL animals had a substantial increase in B cells in fraction F compared to controls, and only a slight decrease was observed in IgHEL+HOD+ mice compared to IgHEL animals (Figure 2D). Correlating with a decrease in mature B cells in SwHEL+HOD+ mice, the percentage of B cells reactive with HEL-tet was decreased 4-fold, compared to SwHEL [Figure 2E, average percentages: SwHEL+ (48%) and SwHEL+HOD+ (12%)]. Similarly, the percentage of B cells reactive with HEL was decreased 2.5-fold in IgHEL+HOD+ mice, compared to IgHEL [Figure 2E, average percentages: IgHEL (88%) and IgHEL+HOD+ (35%)]. Enumeration of HEL-reactive B cells in Fraction F revealed a reduction of 6.8-fold in SwHEL+HOD+ and 5.4-fold in IgHEL+HOD+ mice compared to their respective BCR-Tg control mice [Figure 2F, mean HEL-reactive B cell numbers: SwHEL+ (6,789), SwHEL+HOD+ (998), IgHEL+ (22,172), and IgHEL+HOD+ (4,123)]. Thus, central tolerance mechanisms in both IgHEL+HOD+ and SwHEL+HOD+ mice decreased the numbers of autoreactive B cells to an RBC-specific antigen. However, while SwHEL+HOD+ mice appeared more efficient than IgHEL+HOD+ mice in decreasing HEL-specific B cells, mature autoreactive B cells were observed in both strain combinations.

### HEL-Reactive Marginal Zone (MZ) B Cells Are Enriched in Autoreactive IgHEL Mice

**Figure 3:**
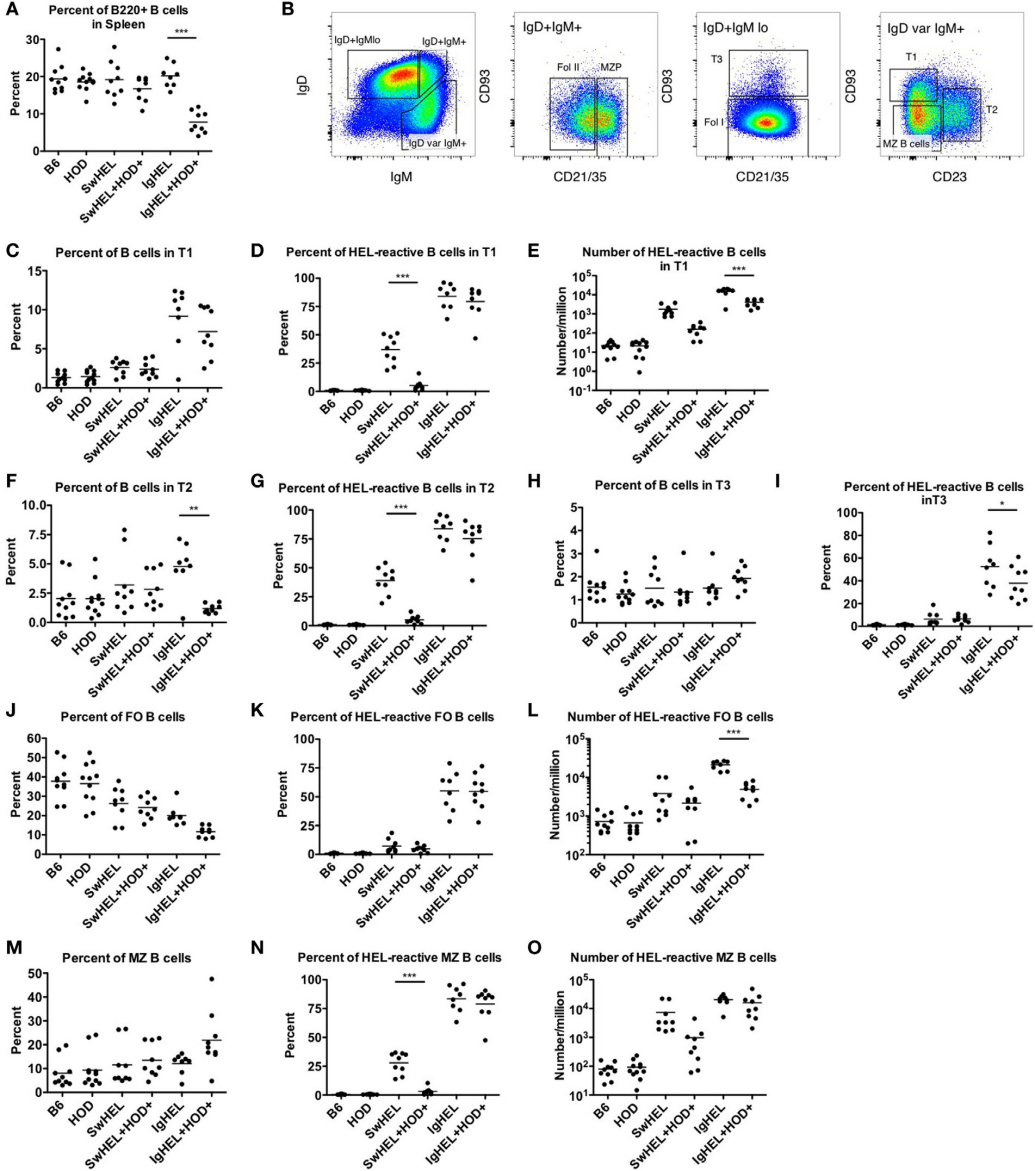
Effect of red blood cell autoantigen on B cell transition and maturation. Splenocytes were harvested from mice and stained with antibodies against IgD, IgM, CD93, CD21/35, CD23, and B220 to define the stages of B cell development in the spleen. To evaluate reactivity to hen egg lysozyme (HEL), cells were stained with albumin-tet to control for nonspecific binding followed by HEL-tet. **(A)** The percentage of B220+ splenic B cells was determined and **(B)** the stages of B cell maturation was outlined. The **(C)** percentage of B cells, **(D)** frequency, and **(E)** absolute number of HEL-reactive B cells was computed for transitional stage 1 (T1). The **(F)** percent and **(G)** number of HEL-reactive B cells was assessed for transitional stage 2 (T2). Similarly, the percent of **(H)** total B cells and **(I)** HEL-reactive B cells was determined for transitional stage 3 (T3). Likewise, the **(J)** percentage of FO B cells, and **(K)** frequency, and **(L)** number of HEL-reactive mature FO B cells were evaluated. The **(M)** percent of MZ B cells was calculated and HEL-reactive MZ B cell **(N)** percentages and **(O)** numbers were determined. Flow plots in **(B)** are representative data from three independent experiments with at least three mice per group. Scatter dot plots in **(A)** and **(C–O)** are compiled data from three independent experiments with at least three mice per group. Selective *p* values are shown on graphs and * ≤ 0.05, ** ≤ 0.01, and *** ≤ 0.001. For complete statistical analysis with all significant differences, see Table S3 in Supplementary Material.

The extent to which peripheral tolerance mechanisms act upon the mature autoreactive B cells detected in SwHEL+HOD+ and IgHEL+HOD+ mice was investigated by analyzing autoreactive splenic B cells that escaped central tolerance. IgHEL+HOD+ mice had significantly fewer splenic B cells, compared to all other groups, including IgHEL (Figure 3A; Table S3 in Supplementary Material). By contrast, no decrease in total B cells was detected in SwHEL+HOD+ mice compared to SwHEL mice. Once B cells leave the bone marrow, they pass though several transient transitional stages (T1, T2, and T3) in the spleen, which serve to dictate the final B cell maturation into follicular (FO) or MZ B cells (representative gating strategy shown, Figure 3B). There was no significant change in the percentage of T1 B cells in SwHEL+HOD+ mice compared to SwHEL mice, both of which were likewise similar to control B6 and HOD mice (Figure 3C). Analysis of the HEL specificity of B cells in these populations demonstrated that the percentage of HEL-specific B cells in T1 in SwHEL mice was significantly decreased in SwHEL+HOD+ mice (Figure 3D). Analysis of absolute numbers showed a similar pattern of decrease in HEL-reactive B cells in T1 in SwHEL+HOD+ compared to SwHEL, although to a lesser extent than predicted by percentage (Figure 3E). A similar trend was observed with percentages of total and HEL-reactive B cells in T2 (Figures 3F,G). The percentages of total and HEL-reactive B cells in T3 for SwHEL and SwHEL+HOD+ were similar to controls (Figures 3H,I). As T3 B cells have been correlated with anergy (23, 24), these findings make it unlikely that a large number of cells are arrested in an anergic T3 state in SwHEL+HOD+ mice.

In contrast to SwHEL, the percentage of B cells was significantly increased in T1 and T2 for IgHEL mice, compared to controls (Figures 3C,F, respectively). In the presence of HEL autoantigen, IgHEL+HOD+ mice had percentages of B cells in T1 similar to IgHEL. However, there was a significant decrease in the percentage of B cells in T2 compared to IgHEL (Figure 3F). Within T1 and T2, the frequency of HEL-reactive B cells was similar between IgHEL and IgHEL+HOD+ (Figures 3D,G) while the absolute number of HEL-reactive B cells in T1 and T2 were decreased in IgHEL+HOD+, compared to IgHEL (Figure 3E and data not shown). Together, these data suggest the peripheral tolerance mechanisms were initiated between T1 and T2. Coupled with the overall decrease of B cells observed in IgHEL+HOD+ mice but a similar percentage of HEL-reactive B cells within each transitional stage, it is hypothesized that clonal deletion occurred. Lastly, there were similar percentages of B cells in T3 from IgHEL mice and IgHEL+HOD+ mice (Figure 3H).

Functionally mature B cells in the spleen comprise FO and MZ B cell subsets; FO B cells localize in primary and secondary lymphoid follicles and require T cell help to secrete antibodies, class-switch, and establish memory B cells. SwHEL and SwHEL+HOD+ mice had statistically significant decreases in the percentage of FO B cells, compared to controls (Figure 3J). In addition, the B cells that comprised the FO B cell compartment were largely not HEL-reactive (autoreactive) (Figure 3K), which is consistent with previous data demonstrating that autoreactive B cells are excluded from participating in germinal center reactions (25, 26). Similarly, IgHEL and IgHEL+HOD+ had a statistically significant reduction in the percent of FO B cells (Figure 3J). However, the frequency of HEL-reactive B cells within FO remained high (Figure 3K, mean 55% for both groups). Again, correlating with the overall decreased numbers of B cells in IgHEL+HOD+ splenocytes, the absolute number of HEL-reactive B cells in FO were significantly reduced compared to IgHEL (Figure 3L).

Marginal zone B cells play a key role in early adaptive immune responses and share many similarities with B-1 B cells, including expression of a poly-reactive BCR and T cell-independent antibody secretion. SwHEL and SwHEL+HOD+ mice had similar frequencies of innate-like MZ B cells, compared to controls; however, the percent and absolute number of HEL-reactive MZ B cells was decreased in SwHEL+HOD+ autoantigen-expressing mice (Figures 3M–O). By contrast, while the percentage of MZ B cells in IgHEL was similar to controls, IgHEL+HOD+ had an increased frequency of MZ B cells (Figure 3M). IgHEL mice had a significant increase in percentage and number of HEL-reactive MZ B cells, compared to controls. However, despite HOD autoantigen expression, the frequency and number of autoreactive MZ B cells in IgHEL+HOD+ mice remained unaltered (Figures 3N,O, respectively).

In aggregate, these data demonstrate that SwHEL BCR-Tg mice follow a similar trend for B cell development as control mice but IgHEL BCR-Tg mice do not. IgHEL BCR-Tg mice had an abnormally high percentage of B cells in transitional stages, which result in decreased frequencies of mature B cells. In the presence of HOD, SwHEL B cells were reduced in each developmental stage and in the mature subsets of B cells. By contrast, autoreactive IgHEL B cells were increased in number in the MZ B cell compartment, a subtype that is most similar to B-1 B cells.

### Autoantibodies Induce Anemia in IgHEL+HOD+ Mice But Not SwHEL+HOD+ Mice

**Figure 4:**
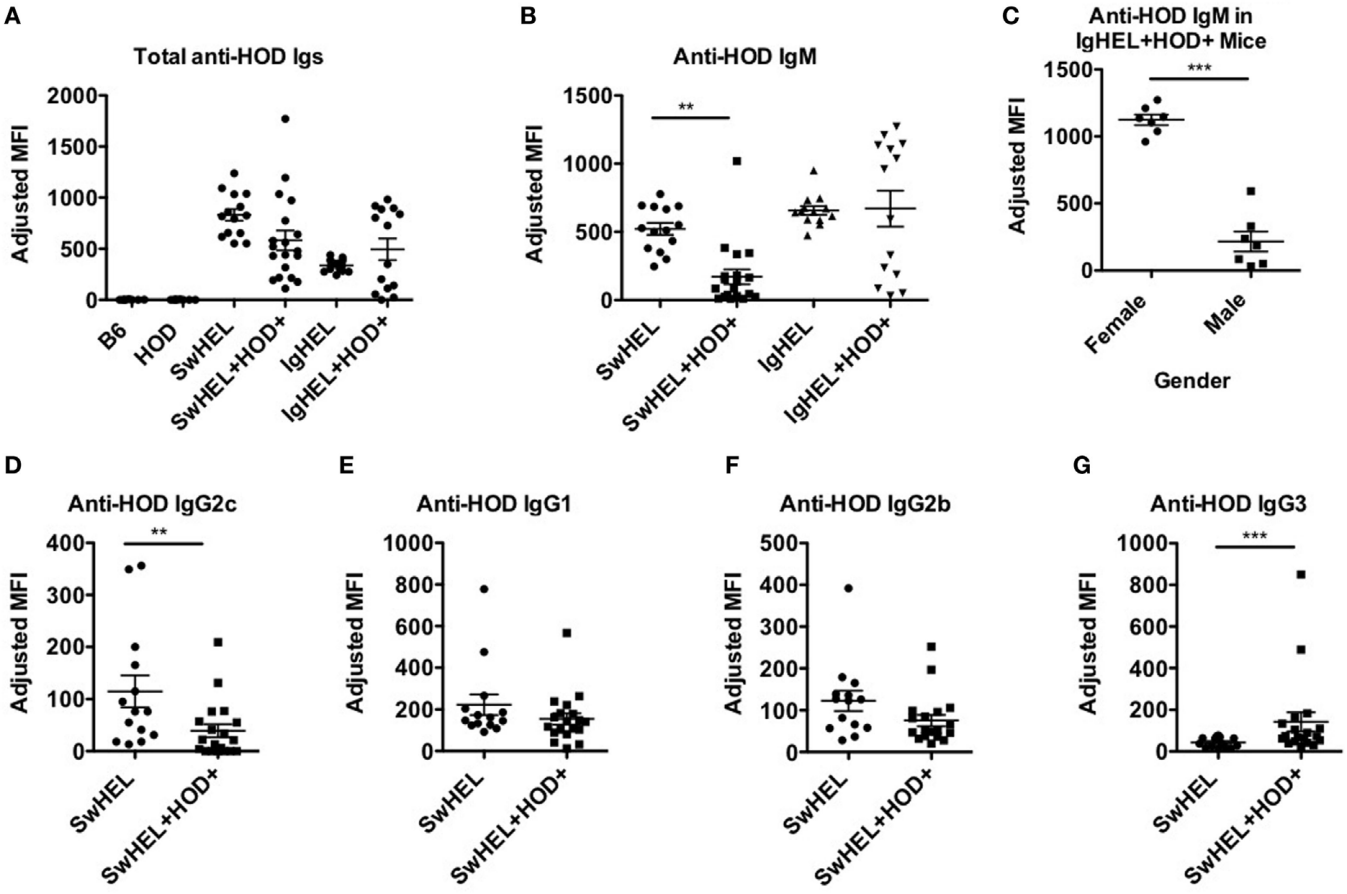
Autoantibodies are detected in SwHEL+HOD+ and IgHEL+HOD+ mice. To evaluate whether B cells that escaped central and peripheral tolerance secreted autoantibodies, sera was collected from experimental mice and assayed for anti-HOD antibodies. **(A)** Total anti-HOD immunoglobulins (Igs) was determined by flow crossmatch against HOD and control B6 red blood cell targets. Anti-HOD IgM antibodies were evaluated in **(B)** BCR-Tg mice and **(C)** the role of gender in autoantibody production was assessed in IgHEL+HOD+ mice. Production of **(D)** IgG2c, **(E)** IgG1, **(F)** IgG2b, and **(G)** IgG3 subtype-specific antibodies were evaluated in SwHEL and SwHEL+HOD+ mice. Scatter dot plots shown are compiled from three independent experiments with at least three mice per group. Selective *p* values are shown on graphs and * ≤ 0.05, ** ≤ 0.01, and *** ≤ 0.001. For complete statistical analysis with all significant differences, see Table S4 in Supplementary Material.

To assess whether tolerance mechanisms employed in SwHEL+HOD+ and IgHEL+HOD+ mice were sufficient to prevent autoantibody production, sera was collected and assessed for anti-HOD autoantibodies by flow crossmatch. Briefly, experimental sera was tested for binding to control B6 and experimental HOD RBCs, as previously described in Ref. (27). No HOD-specific antibodies were detectable in control B6 or HOD mice (Figure 4A; Table S4 in Supplementary Material). Both SwHEL and IgHEL BCR-Tg mice had detectable anti-HOD antibodies in their sera. As a consequence of expression of the HOD antigen on RBCs, total anti-HOD antibodies decreased in SwHEL+HOD+ mice. Further analysis of antibody isotype and subtype revealed that SwHEL+HOD+ mice had significantly less circulating HOD-specific IgM and IgG2c antibodies compared to SwHEL BCR-Tg mice (Figures 4B,D). Similarly, anti-HOD IgG1 and IgG2b antibody subtypes were decreased in SwHEL+HOD+ mice compared to SwHEL BCR-Tg controls, but not significantly whereas anti-HOD IgG3 was slightly increased (Figures 4E–G). By contrast, a bimodal distribution of total anti-HOD autoantibodies was observed in IgHEL+HOD+ mice; elevated autoantibodies were detectable in a subset of IgHEL+HOD+ mice, compared to IgHEL controls (Figure 4A). Similarly, IgHEL+HOD+ mice had a bimodal distribution of anti-HOD IgM autoantibodies (Figure 4B). Given that the frequency of many autoimmune diseases are elevated in females (28, 29), anti-IgM titers in IgHEL+HOD+ mice were compared across gender; female IgHEL+HOD+ mice had significantly higher titers of anti-HOD IgM autoantibodies, compared to their male littermates thereby accounting for the bimodal distribution of autoantibodies (Figure 4C). No HOD-specific IgG antibodies were detected in IgHEL or IgHEL+HOD+ mice, as predicted due to the inability of the transgene to class-switch (data not shown).

**Figure 5:**
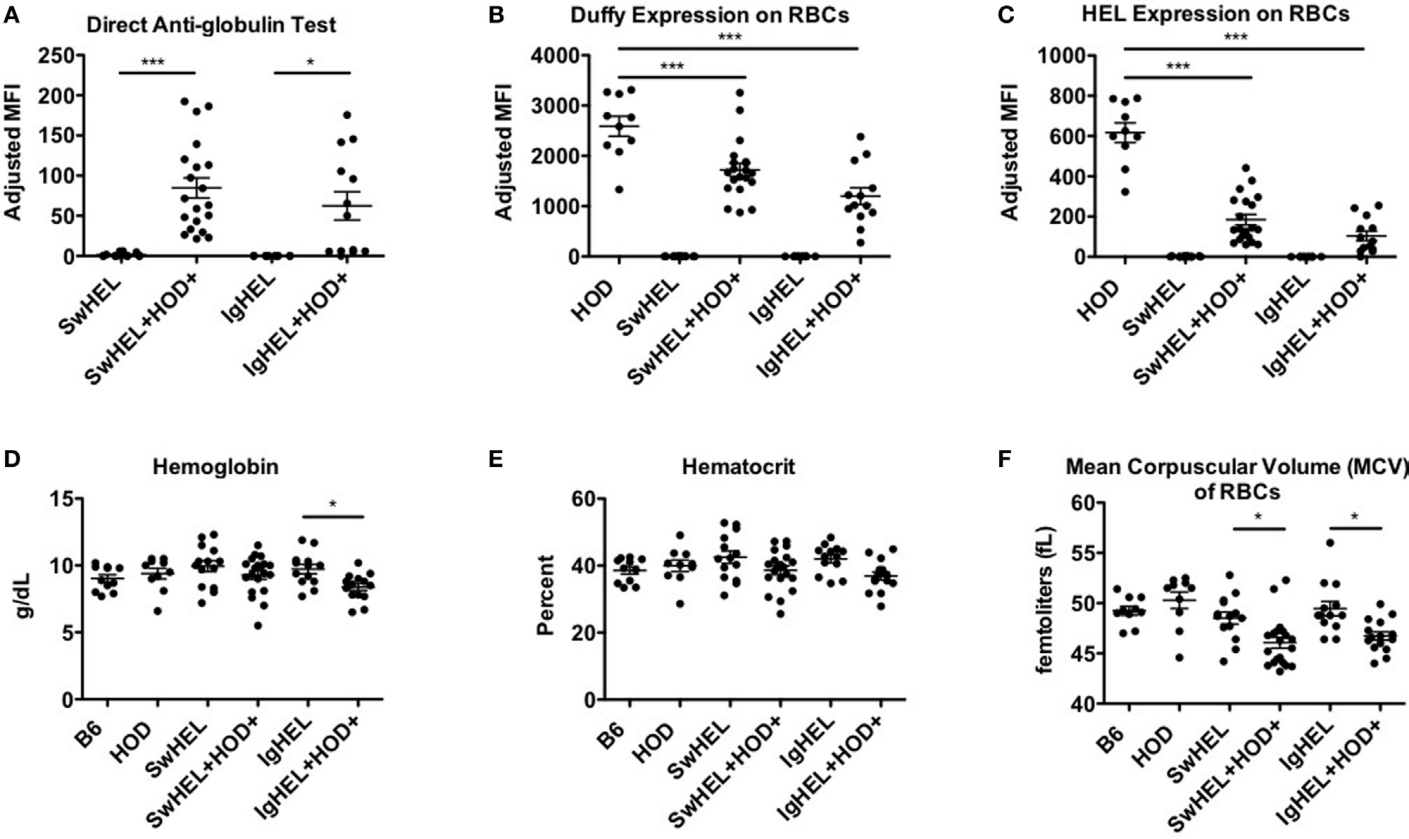
Autoantibodies effect red blood cell (RBCs). To determine whether the presence of autoantibodies affected erythrocytes, RBCs from experimental mice were collected and assayed for **(A)** antibodies bound to their surface and surface expression of **(B)** Duffy and **(C)** hen egg lysozyme (HEL) antigens. To assess whether autoantibodies affect RBCs, a hematology profile was generated with a HemaVet. RBCs from experimental mice were evaluated for **(D)** hemoglobin, **(E)** hematocrit, and **(F)** mean corpuscular volume (MCV). Scatter dot plots shown are compiled from three independent experiments with at least three mice per group. Selective *p* values are shown on graphs and * ≤ 0.05, ** ≤ 0.01, and *** ≤ 0.001. For complete statistical analysis with all significant differences, see Table S5 in Supplementary Material.

The observation that lower levels autoantibodies were detectable in IgHEL+HOD+ compared to SwHEL+HOD+ mice was unexpected due to the enrichment of peritoneal B-1 B cells and incomplete deletion of B-2 HEL-reactive B cells (Figures 1B and 2D–F). Modulation of circulating antibody levels is consistent with autoantibodies bound to their cognate antigen. Thus, to test the hypothesis that anti-HEL autoantibodies were bound to the RBCs in SwHEL+HOD+ and IgHEL+HOD+ mice, direct anti-globulin tests were performed. SwHEL+HOD+ RBCs had significantly more antibodies bound to their surface, compared to SwHEL controls (Figure 5A; Table S5 in Supplementary Material). Similarly, more antibodies were detected on RBCs from IgHEL+HOD+ mice, compared to IgHEL controls. However, there was a bimodal distribution of antibody-bound RBCs, with some IgHEL+HOD+ RBCs not bound by antibodies. Presence of HOD-specific antibodies but absence of bound antibodies on RBCs suggest that circulating RBCs may not express the HOD antigen. Antigen loss is a well-described antibody-mediated phenomenon that occurs frequently to RBC antigens whereby RBCs cease to express that particular antigen and cannot re-synthesize new protein due to the absence of a nucleus (30). Thus, to test the hypothesis that expression of the HOD antigen is modulated in SwHEL+HOD+ and IgHEL+HOD+ mice, RBCs were stained with anti-Duffy and anti-HEL antibodies to detect components of the HOD antigen. RBCs from both SwHEL+HOD+ and IgHEL+HOD+ had significant reductions in the expression of both Duffy and HEL components of the HOD antigen, compared to naïve HOD mice, suggesting HOD antigen loss has occurred (Figures 5B,C).

Some RBC-specific antibodies can be pathogenic and lead to hemolysis. To determine whether the autoantibodies detected in SwHEL+HOD+ and IgHEL+HOD+ were pathogenic thereby resulting in anemia, whole blood samples were analyzed. No significant differences were detected in total RBC counts in any experimental group (data not shown). SwHEL+HOD+ mice had comparable levels of hemoglobin and hematocrit as B6, HOD, and SwHEL controls (Figures 5D,E), but decreased RBC mean corpuscular volume (MCV) (Figure 5F). By contrast, IgHEL+HOD+ mice had decreased hemoglobin, hematocrit, and MCV, compared to IgHEL BCR-Tg and control animals. Thus, taken together, RBCs in autoreactive IgHEL+HOD+ mice have altered hematological parameters consistent with anemia, which was not observed in SwHEL+HOD+ animals. In aggregate, these data suggest that the pathogenicity of RBC-specific autoantibodies is influenced by the ability of B cells to undergo class-switching; by extension, the method utilized in generating BCR-Tg mice (random integration vs. knock-in) may bias subsequent immune tolerance and responses.

## Discussion

The current understanding of tolerance to RBC-specific antigens has largely been generated using the autoAb 4C8 BCR-Tg mouse model, which has provided substantial data to support a paradigm in which B-1 B cells are the source of autoantibodies in AIHA (5–9). However, the autoAb 4C8 BCR-Tg mouse was constructed using a fully rearranged IgM, regulated by recombinant elements, and located outside the natural Ig locus due to random integration from pro-nuclear injection. While this approach was widely used in the first generation of BCR transgenics, it has since been appreciated that more physiological data are obtained through homologous recombination of VDR domains into Vh regions of the Ig locus (knock-in BCR mice), to allow normal receptor editing and class-switching. In this report, we directly compare a random integrant vs. a knock-in BCR-Tg mouse in a model of RBC autoreactivity. In order to control for other variables, both BCR mice utilize the same VDR (anti-HEL HyHEL10) and are crossed with the same mouse expressing HEL epitopes on RBCs (HOD mouse) (6, 17, 18). In mice with intact receptor editing (SwHEL+HOD+ mice), autoreactive B-1 B cells were reduced to almost background levels, whereas autoreactive B-2 B cells were likewise decreased but to a lesser extent. By contrast, in mice with the inability to receptor edit (IgHEL+HOD+), there was a selective enrichment of B-1 B cells in the peritoneum and splenic MZ B cells, and a concomitant decrease in B-2 B cells. Moreover, anti-HOD autoantibodies are detected in both SwHEL+HOD+ and IgHEL+HOD+ mice, but autoantibodies significantly affect RBCs and may cause anemia only in IgHEL+HOD+ mice. Thus, these data demonstrate that the genetic approach to generate BCR-Tg mice significantly impacts the B cell repertoire and may not accurately recapitulate normal immune education and tolerance. Importantly, the existing paradigm for B cell education to RBC antigens rests almost entirely upon the autoAb 4C8 BCR-Tg mouse, which gives similar results to the IgHEL mouse, neither of which receptor edit or class-switch; whereas a very different outcome is observed with the SwHEL mouse, which retains the ability to receptor edit away from autoreactivity and class-switch into additional antibody isotypes and subtypes.

The data obtained from the IgHEL+HOD+ BCR-Tg model are similar to the findings reported by Murakami and colleagues with the autoAb 4C8 BCR-Tg system (10, 11). Of note, because the RBC antigen recognized by the autoAb 4C8 BCR-Tg mouse is essentially conserved in all known strains, the autoAb 4C8 BCR system has no control to study baseline B cells in the absence of autoantigen. In IgHEL BCR-Tg mice alone (no autoantigen), HEL-reactive B cells were detectable in secondary lymphoid organs and the peritoneal cavity demonstrating that there was not a preferential development of the B-1 B cell compartment at baseline. In addition, IgHEL B cells were observed in all stages of B cell development in the bone marrow and spleen. However, due to expression of a “fixed” BCR, there were significant increases in the numbers of IgHEL B cells in the bone marrow and transitional stages of B cell development in the spleen when compared to controls (Figures 2D and 3C,F). This could be attributed to the forced expression of affinity matured IgM and IgD, the inability to undergo class-switching, or an altered regulation of expression of cell surface markers utilized to delineate subtype.

Analysis of IgHEL+HOD+ mice demonstrated that the absolute numbers of autoreactive HEL-specific B-2 cells are reduced in the bone marrow and spleen (Figures 2A and 3A). This reduction is most likely due to clonal deletion, which has been observed in other BCR-Tg autoreactive models (31, 32). In contrast to the conventional B-2 B cells, there was a selective enrichment of B-1 B cells in the peritoneum. The increase of autoreactive B-1 B cells could be due to lack of exposure to the autoantigen due to development in extramedullary compartments. However, the substantial reduction of autoreactive B-2 B cells in the peritoneum, presumably due to encountering autoantigen, may argue against this notion (Figure 1C, right). Of course, we cannot rule out autoreactive B-2 but not B-1 B cells trafficking out of peritoneum to encounter autoantigen (thus prompting deletion), but B-1 B cells also have the capacity to migrate to and from the spleen (33, 34). An alternative explanation is that B-1 B cells have encountered autoantigen, but due to the inherent properties of B-1 B cells, they undergo activation and proliferation instead of deletion (35). This is plausible, as developmentally, newly forming B cells that receive a strong signal upon encounter with autoantigen are selected into the B-1 B cell compartment; B-2 B cells are more sensitive to ligation with autoantigen and are instructed to rearrange their BCR, undergo deletion, or become anergic.

In the SwHEL+HOD+ mouse, B-1 B cells are not preferentially enriched; on the contrary, autoreactive HEL-specific B cells are reduced to background levels (Figure 1B, right). Similar to IgHEL+HOD+ mice, numbers of autoreactive B-2 B cells in the peritoneum are significantly decreased (Figure 1C, right), suggesting that B-1 and B-2 B cells are capable of being tolerized upon autoantigen encounter. As such, we hypothesize that receptor editing plays a substantial role in tolerizing autoreactive B cells, as the total numbers of B cells in each population analyzed from SwHEL and SwHEL+HOD+ mice were similar to controls, but the frequency and number of autoreactive HEL-reactive B cells were significantly reduced in HOD+SwHEL+ mice. However, despite reductions in autoreactive B cells, autoantibodies were detectable in SwHEL+HOD+ mice. Autoantibody production in SwHEL+HOD+ mice was not predicted as Phan et al. observed anergy and significant reductions in autoantibodies in SwHEL mice that were bred with soluble HEL animals (18). Thus, these data further illustrate that the type of autoantigen may also influence B cell tolerance mechanisms.

An additional unexpected finding in our studies was a bimodal distribution of anti-IgM autoantibodies in IgHEL+HOD+ mice. Previously, Phan et al. demonstrated significant reductions of autoantibodies in IgHEL mice bred with soluble HEL animals (18). However, when IgHEL mice were bred with HOD mice, which express membrane-bound HEL antigen, a subset of IgHEL+HOD+ mice made higher titers of IgM autoantibodies compared to IgHEL controls. Upon further investigation, our data revealed sex-based differences in autoantibody production in IgHEL+HOD+ mice whereby females made significantly higher titers of anti-HOD IgM compared to their male littermates (Figure 4C). Despite the disparity in autoantibody titers, no significant sex-based differences were observed in other RBC parameters evaluated, such as hemoglobin, hematocrit, or MCV (data not shown). Moreover, the presence of autoantibodies did not affect B cell development as similar frequencies of total and HEL-reactive B cells in Fraction F of the bone marrow, splenic MZ, and FO B cells were observed between female and male IgHEL+HOD+ mice (data not shown). By contrast, no gender-based differences were observed in SwHEL+HOD+ mice. While a female predilection for autoimmunity has been described in many autoimmune models, it is unclear why a gender bias was observed in IgHEL but not SwHEL crosses. Regardless, these data further draw a distinction between data obtained with the two BCR-Tg mice.

Taken together, these data reject the hypothesis that there is a single population or subset of B cells that serve as a reservoir for RBC-specific autoreactive B cells. Instead, the data suggest a more complex model, in which several phenotypes of B cells persist in the bone marrow and periphery despite tolerance mechanisms. Thus, these findings challenge the current paradigm that views B-1 B cells as the dangerous population of autoreactive B cells with respect to RBC antigens. Such a challenge also questions efforts to generate therapeutics for AHIA (either prophylactic or interventional) that specifically target B-1 B cells. Our findings demonstrate the necessity of a conceptual re-evaluation of B cell tolerance to RBC antigens and ongoing studies into baseline tolerance to RBCs, and the mechanisms by which it is lost in the pathogenesis of AIHA.

## Materials and Methods

### Mice

C57BL/6 (B6) and IgHEL mice were purchased from Jackson Laboratories (Bar Harbor, ME, USA). IgHEL mice are listed as MD4 animals; IgHEL has been used in the current paper for clarity of nomenclature. SwHEL mice were a kind gift from Dr. Robert Brink. HOD (with RBC-specific expression of HEL, a portion of ovalbumin, and the human blood group antigen Duffy), IgHEL (also known as MD4), and SwHEL mice were bred at Bloodworks NW Vivarium. Mice were maintained on standard rodent chow and water in a temperature- and light-controlled environment and used at 8–12 weeks of age. All experiments were performed according to approved Bloodworks Northwest Institutional Animal Care and Use Committee (IACUC) procedures.

### Tetramerization of HEL and Albumin

Hen egg lysozyme (Sigma) was resuspended to a concentration of 1 mg/mL in PBS and biotinylated with the EZ-Link Sulfo-NHS Biotinylation Kit according to the manufacturer’s recommendations (Pierce). Albumin (Sigma) was similarly biotinylated. Both HEL and albumin were tetramerized with 10 additions of APC (Molecular Probes) and APC-Cy7 (Molecular Probes), respectively.

### Staining Leukocytes

Bone marrow, splenocytes, and peritoneal cavity cells were harvested. RBCs were lysed with lysis buffer (Sigma). Leukocytes were stained with Albumin-APC-Cy7 for 30 min on ice followed by HEL-APC (HEL-tet). After cells were stained with both tetramers, organ-specific surface antibodies were added. Bone marrow cells were stained with antibodies against B220, CD43, BP-1, IgM, IgD, CD24, and CD93. Splenocytes were stained with antibodies directed against IgM, IgD, CD93, CD23, B220, CD19, and CD21/35. Peritoneal leukocytes were stained with antibodies against CD3, CD11b, CD5, IgD, IgM, F480, and CD43. All antibodies were purchased from eBioscience. Absolute cell counts were determined by use of APC beads (BD Biosciences). Cells were washed with FACS buffer [PBS + 0.2 mg/mL bovine serum albumin (Sigma) + 0.9 mg/mL EDTA (Sigma)] and analyzed on an LSRII flow cytometer.

### Antibody Detection and RBC Analysis

Flow crossmatch and direct anti-globulin test were performed as previously described (27, 36). Isotype (IgM and IgG) and subtype-specific (IgG1, IgG2b, IgG2c, and IgG3) were utilized. To assess for HOD antigen expression, RBCs were stained with monoclonal antibodies specific for HEL (4B7) and Duffy (MIMA-29), as previously described (17, 20). Hemogram data on whole blood was collected with a HemaVet Hematology Analyzer (Drew Scientific).

### Statistical Analysis and Graphing

Statistical significance for two groups was determined by an unpaired Student’s *T*-test, whereas multiple groups were evaluated by a one-way ANOVA followed by a Bonferroni’s multiple test comparison post-test was utilized for three or more groups. Significance was set at *p* ≤ 0.05 and * ≤ 0.05, ** ≤ 0.01, *** ≤ 0.001. *P* values for selective groups are shown on graphs but for a more detailed and complete list of statistically significant differences, please refer to the Supplementary Tables. Flow plots were generated using Flow Jo software (TreeStar) and graphs were generated using Graphpad Prism (La Jolla, CA, USA).

## Ethics Statement

All experiments were performed according to approved BloodworksNW Institutional Animal Care and Use Committee (IACUC) procedures.

## Author Contributions

AR, HH, LK, JH, JZ, and KH each performed experiments and analyzed data contained in this work. KH and JZ authored the manuscript and all authors read, corrected, and approved of the final manuscript.

## Conflict of Interest Statement

AR, HH, LK, JH, and KH have no conflicts of interest to declare. JZ serves on the Scientific Advisory Board for Rubius Therapeutics and consults for Surface Oncology and Sinopia Biosciences.

## Supplementary Material

Table S1 **Complete statistical analysis for data Figure 1**.

Table S2 **Complete statistical analysis for data Figure 2**.

Table S3 **Complete statistical analysis for data Figure 3**.

Table S4 **Complete statistical analysis for data Figure 4**.

Table S5 **Complete statistical analysis for data Figure 5**.
